# Endoscopic Ultrasound-Guided Lumen-Apposing Metal Stent Drainage in Benign Pancreatobiliary and Gastrointestinal Disease: Evolving Techniques and Clinical Outcomes

**DOI:** 10.3390/diagnostics16040522

**Published:** 2026-02-09

**Authors:** Filippo Antonini, Marco Valvano, Edoardo Troncone, Domenico Galasso, Amedeo Montale, Mario Capasso, Matteo Marasco, Benedetto Mangiavillano, Giovanna Del Vecchio Blanco, Mauro Dalla Libera, Antonella Scarcelli, Antonio Facciorusso, Lorenzo Fuccio, Massimiliano Mutignani, Manuel Perez-Miranda

**Affiliations:** 1 Gastroenterology and Interventional Endoscopy Unit, C. e G. Mazzoni Hospital, 63100 Ascoli Piceno, Italy; 2 Division of Gastroenterology, Galliera Hospital, 16128 Genoa, Italy; 3 Gastroenterology Unit, Division of Gastroenterology, Hepatology, and Nutrition, Department of Life, Health and Environmental Sciences, University of L’Aquila, 67100 L’Aquila, Italy; 4 Department of Systems Medicine, University of Rome “Tor Vergata”, 00133 Rome, Italy; 5 Gastroenterology Unit, Internal Medicine Service, Hopital Riviera-Chablais, 1847 Rennaz, Switzerland; 6 Gastroenterology and Endoscopy Unit, ASST Ospedale Maggiore, 26013 Crema, Italy; 7 Gastroenterology and Digestive Endoscopy Unit, Santa Rosa Hospital, 01100 Viterbo, Italy; 8 Department of Medical and Surgical Sciences and Transitional Medicine, Sapienza University, 00161 Rome, Italy; 9 Gastrointestinal Endoscopy Unit, Humanitas Mater Domini, 21053 Castellanza, Italy; 10 Digestive Endoscopy Unit, ASL 2 Savonese, 17100 Savona, Italy; 11 Division of Gastroenterology, “San Salvatore” Hospital, 61121 Pesaro, Italy; 12 Gastroenterology Unit, Faculty of Medicine and Surgery, University of Salento, 73100 Lecce, Italy; 13 Gastroenterology Unit, Department of Medical and Surgical Sciences, IRCCS Azienda Ospedaliero-Universitaria di Bologna (IRCCS University Hospital of Bologna), University of Bologna, 40125 Bologna, Italy; 14 Endoscopy Unit, ASST Grande Ospedale Metropolitano Niguarda, 20162 Milan, Italy; 15 Department of Gastroenterology and Hepatology, University Hospital Rio Hortega, 47012 Valladolid, Spain

**Keywords:** endoscopic ultrasound, LAMS, biliary drainage, EUS-guided drainage, gallbladder drainage, gastric outlet obstruction, EUS-directed transgastric ERCP, EDGE, gastroenterostomy

## Abstract

Interventional endoscopic ultrasound (EUS) has become a cornerstone in the management of malignant pancreatobiliary diseases, offering minimally invasive alternatives to traditional surgical approaches. More recently, accumulating evidence supports its expanding role in the treatment of benign pancreatobiliary conditions, including acute cholecystitis and pancreatitis, benign gastric outlet obstruction, and scenarios involving altered gastrointestinal anatomy. This narrative review provides an overview of key EUS-guided drainage techniques utilizing lumen-apposing metal stents (LAMSs) in benign settings. It focuses on procedures such as EUS-guided gallbladder drainage, drainage of abdominal collections, EUS-directed transgastric ERCP (EDGE), and EUS-gastroenterostomy. These interventions have demonstrated high technical and clinical success rates, favorable safety profiles, and expanding indications, particularly among patients who are poor surgical candidates. This review highlights evolving techniques, clinical outcomes, and the impact of device innovations on procedural efficacy and safety.

## 1. Introduction

Interventional endoscopic ultrasound (EUS) has emerged as a transformative modality in the management of gastrointestinal and pancreatobiliary diseases [1,2]. Over the years, EUS has introduced a variety of innovative applications, with EUS-guided drainage standing out as a key advancement. This technique has significantly revolutionized the management of numerous conditions, offering a safer, less invasive alternative to traditional surgical approaches and interventional radiology [3,4,5]. The success of EUS-guided drainage procedures relies on several fundamental principles, including precise access to target areas, the use of specialized devices, and the integration of innovations like lumen-apposing metal stents (LAMSs) [6,7]. These technological advancements have not only improved procedural efficacy but have also expanded the indications and clinical outcomes of EUS-guided drainage, solidifying it as an essential technique for managing complex pancreatobiliary diseases [8,9].

A key aspect of this evolution is the increasing overlap between EUS-guided interventions and traditional endoscopic retrograde cholangiopancreatography (ERCP) techniques. The growing capabilities of EUS, particularly in biliary interventions, have brought it closer to ERCP in terms of procedural competence [10,11]. As a result, interventional endoscopists are now required to be proficient in both EUS and ERCP, as many procedures demand a seamless integration of both techniques to optimize patient outcomes [11].

Initially developed and widely applied for malignant conditions, the role of interventional EUS in the treatment of benign diseases has rapidly evolved [12]. This shift is largely due to ongoing innovations in endoscopic techniques, the development of more refined devices, and a growing body of clinical evidence supporting its effectiveness [13,14].

This narrative review provides an in-depth exploration of the evolving techniques and clinical outcomes of EUS-guided drainage using LAMSs, focusing on benign indications such as acute cholecystitis, pancreatic fluid collections, gastric outlet obstruction of benign etiology, and management in patients with surgically altered gastrointestinal anatomy. By examining these advances, this review highlights the expanding role of EUS in the treatment of benign pancreatobiliary conditions and its growing significance in modern clinical practice.

## 2. Technical Foundations of EUS-Guided Drainage

### 2.1. EUS-Guided Access Principles

The fundamental principle behind EUS-guided drainage is to achieve safe and precise access to target fluid collections or bile ducts using endoscopic ultrasound as a guiding tool [15,16]. Under EUS guidance, the endoscope is positioned within the gastrointestinal tract, typically the stomach or duodenum, to allow real-time imaging of adjacent structures [15,16]. Using this imaging, the physician identifies the optimal location for accessing the target area, such as a gallbladder, pancreatic fluid collection, or biliary duct [15,16].

The typical approach involves puncturing the gastrointestinal wall with a fine needle (usually a 19-gauge aspiration needle) and then advancing a guidewire through the needle into the target structure. The guidewire allows for subsequent dilation and stent placement. This access is often facilitated through the use of echoendoscopes, which combine both ultrasound and endoscopic visualization, enabling accurate assessment of tissue depth and avoiding injury to surrounding organs and vasculature [15,16].

### 2.2. LAMS: Structure and Function

LAMSs have become a cornerstone of EUS-guided drainage due to their unique structure and design, which facilitate a more secure and durable drainage [2,17,18]. LAMSs are specialized stents designed with a flared, accordion-like structure that allows for apposition of two lumens (e.g., the gallbladder and the stomach or pancreatic collection and the duodenum). Their self-expanding design ensures a secure fit, reducing the risk of stent migration and allowing for long-term drainage [2,17]. One of the key advantages of LAMSs over traditional plastic stents is their ability to provide a larger drainage lumen, which is particularly useful in draining larger fluid collections or achieving decompression in complex anatomical settings. The unique design of LAMSs also helps to minimize the risk of leakage and bile peritonitis, common concerns in traditional drainage procedures [2,17]. Their high technical success rates, particularly in cases involving biliary and pancreatic collections, have been well documented in clinical studies [17,19].

An important innovation in EUS-guided drainage is the development of electrocautery-enhanced LAMSs (EC-LAMSs) [17]. These stents are equipped with a built-in electrocautery mechanism that allows for controlled tissue puncture and dilation during the procedure. The advantage of EC-LAMSs over traditional LAMSs is the ability to perform puncture and stent deployment in one continuous motion, reducing the need for multiple steps and minimizing procedure time [19,20].

The electrocautery mechanism works by delivering a high-frequency electrical current to the tissue, which facilitates easy penetration of the gastrointestinal wall. This feature is particularly beneficial in cases where the tissue is dense or scarred, such as in patients with chronic pancreatitis or those who have undergone previous surgeries. EC-LAMSs have improved technical success rates and reduced the likelihood of adverse events such as bleeding or perforation. The shorter procedural time and the reduced need for multiple instrument exchanges make EC-LAMSs an appealing choice for clinicians and patients alike [21].

### 2.3. Patient Selection and Procedural Planning

Patient selection is a critical component of successful EUS-guided drainage. Ideal candidates are those with conditions who require drainage but are not suitable candidates for traditional surgical interventions due to factors like comorbidities, frailty, or poor surgical prognosis. Conditions such as cholestatic jaundice, acute cholecystitis, pancreatic fluid collections, and gastric outlet obstruction are common indications for EUS-guided drainage [21,22]. Additionally, patients with altered gastrointestinal anatomy, such as those who have undergone Roux-en-Y gastric bypass or other reconstructive surgeries, may benefit from these techniques as they present challenges for conventional surgery [23].

Pre-procedural planning involves a thorough assessment of the patient’s medical history, current health status, and imaging studies [24,25]. The physician must evaluate the size, location, and nature of the collection or obstruction to determine the most appropriate technique and device [1,24]. Factors such as the patient’s anatomy and comorbid conditions also guide the decision-making process, ensuring the procedure is both technically feasible and safe [1,26].

## 3. EUS-Guided Drainage of Pancreatic and Peripancreatic Collections

Pancreatic and peri-pancreatic collections (PCs) typically arise from complicated acute pancreatitis or, alternatively, as a result of pancreatic or abdominal surgery (i.e., post-operative collections) [27,28]. PCs diagnosed within the first 4 weeks from the onset of pancreatitis are defined as acute collections and generally lack a mature wall. After 4 weeks, PCs are classified as either pancreatic pseudocysts (PP) or walled-off necrosis (WON), depending on the amount of solid necrotic material within the collection [29]. This classification is crucial when evaluating both the indication and the technique for PC drainage. PC drainage is usually indicated in presence of symptoms or infection, and the timing from onset, the presence of a mature wall, as well as the extent of necrosis all influence the drainage strategy (percutaneous vs. endoscopic; plastic stents vs. LAMSs) [27,30].

### 3.1. Procedural Considerations and Stent Placement

**Figure 1:**
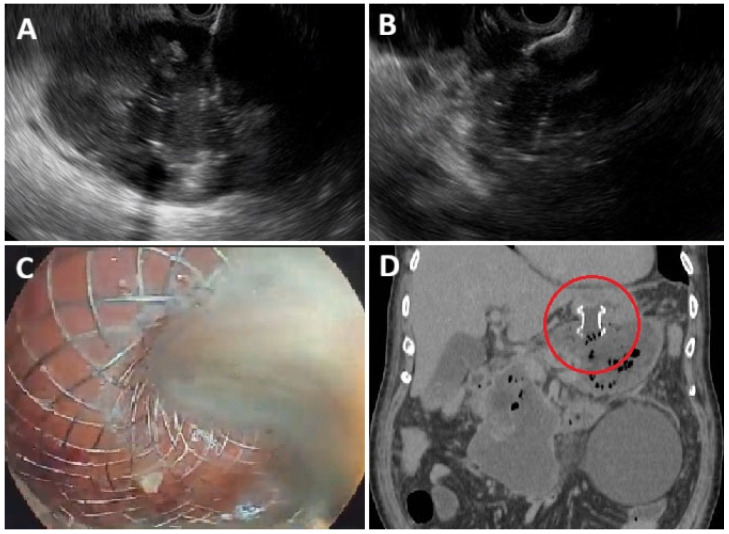
Procedural steps of EUS-guided drainage of a large walled-off necrosis (WON). (**A**) EUS view showing the WON. (**B**) Deployment of a lumen-apposing metal stent (LAMS) within the WON. (**C**) Drainage of purulent material through the LAMS. (**D**) Abdominal CT scan demonstrating a transgastric LAMS in place (red circle) for WON drainage.

**Figure 2:**
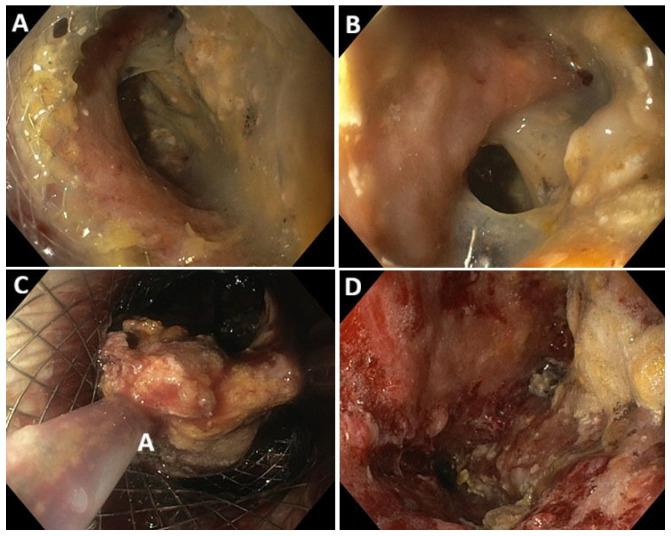
Procedural steps of endoscopic necrosectomy: (**A**,**B**): Endoscopic view of the WON cavity through the LAMS. (**C**): Endoscopic view of direct necrosectomy with a snare through the LAMS. (**D**): Endoscopic view of the cavity after necrosectomy.

Over the years, EUS-guided drainage of PC has gained wide popularity as a minimally invasive therapeutic approach, outperforming surgical and percutaneous interventions in terms of both clinical efficacy and safety [30,31,32,33,34]. The availability of LAMS—particularly the cautery-enhanced versions—has significantly contributed to this trend by offering a user-friendly device that enables the creation of a stable fistula between the collection and the gastrointestinal lumen. Using LAMSs instead of traditional plastic double-pigtail stents (DPSs) for PC drainage offers several theoretical advantages: (1) Rapid EUS-guided stent placement, avoiding the need for multiple device exchanges typical of DPS drainage; (2) Unique structural features that minimize the risk of leakage and migration; (3) A wide caliber that facilitates drainage of large-volume collections or solid debris; (4) A stable access tract for direct endoscopic necrosectomy (EN), without requiring stent removal (Figure 1 and Figure 2).

### 3.2. Outcomes and Safety

Several observational studies have reported excellent outcomes in terms of technical and clinical success as well as safety, particularly in the management of WON [35,36,37,38,39] (Table 1). Moreover, PC drainage with LAMSs has also been reported as safe in selected cases of early drainage (<4 weeks) and for post-operative abdominal collections [40,41].

While the use of LAMSs for PC drainage has demonstrated a favorable safety profile, the AE rate is not negligible and should be carefully considered before the procedure, as surgical or radiological rescue may become necessary [42,43,44]. The overall adverse events (AE) rate is estimated at 7–20%, with the most common events including bleeding, infection, stent migration, perforation, and buried stent [43,44,45]. Recent data also suggest that different LAMS models may carry varying bleeding risks, due to design-specific features, while maintaining similar clinical success rates [44,46].

The placement of a coaxial DPS within the LAMSs has been proposed to reduce AEs, particularly bleeding and obstruction. While retrospective studies have shown conflicting results, a randomized clinical trial (RCT) by Vanek et al. reported a lower AE rate in the coaxial DPS group compared to the LAMS-alone group (20.7% vs. 51.5%, *p* = 0.008), although this was not associated with a significant difference in reintervention rates (29.4% vs. 48.5%, *p* = 0.109) [47,48,49]. A meta-analysis of 9 studies (709 patients) confirmed a reduced risk of stent obstruction (OR 0.59; *p* = 0.004) and infection (OR 0.55; *p* = 0.001), though no significant differences were found in overall AE rates [50]. Therefore, it remains unclear whether this strategy should be adopted routinely [51].

**Table 1:** Summary of the main prospective studies comparing LAMSs and DPSs for endoscopic WON drainage. LAMS, lumen-apposing metal stent; DPS, double pigtail stent.

Author (Year)	Study Design	Population	LAMS	DPS	Primary Outcome	Number of Patients	Clinical Success	Total Procedures or Necrosectomy Sessions	Lenght of Hospital Stay (Days)	Adverse Events	Total Costs
Bang (2019) [52]	RCT	Symptomatic and/or infected WON	Hot Axios 15 mm	2 7 Fr	Number of procedures	LAMS = 31 DPS = 29	93.5% (29/31) 96.6% (28/29)	2.0 (2–7) ^§^ 3.0 (2–7) ^§^	3 (0–38) ^§^ 4 (0–103) ^§^	41.9% (13/31) 20.7% (6/29)	53,117 US$ 50,132 US$
Boxhoorn (2023) [53]	Comparative non-randomized	Infected WON	Hot Axios 15–20 mm	2 7 Fr	Need for Necrosectomy	LAMS = 53 DPS = 51	NR NR	64% (34/53) 53% (27/51)	43 ^+^ 53 ^+^	41.5% (22/53) 43% (22/51)	46,860 € 53,208 €
Karstensen (2023) [54]	RCT	WON > 15 cm	Hot Axios 20 mm	2 7 Fr	Number of Necrosectomies	LAMS = 20 DPS = 22	94.7% (18/20) 95.5% (21/22)	3.1 (3.7) ^+^ 2.2 (3.1) ^+^	58 (40–86) ^§^ 43 (40–67) ^§^	5% (1/20) 20% (4/20)	39,176 € 33,939 €
Gornals (2024) [55]	RCT	Symptomatic WON	Hot Axios 10–15–20 mm	1–2 7–10 Fr	Short Term Clinical Success (4 weeks)	LAMS = 33 DPS = 31	63% (21/33) 45% (14/31)	3.0 (2–3) ^§^ 3.0 (2–4) ^§^	34 (3.7–83.2) ^§^ 38 (14–54) ^§^	36% (12/33) 45% (14/31)	35,145 € 35,488 €
Moon (2024) [56]	RCT	Infected WON	Hot Spaxus 16 mm	1–2 7 Fr	Number of Necrosectomies	LAMS = 23 DPS = 23	59.1% (13/23) 30.4% (7/23)	9.0 (8.0–9.0) ^§^ 4.0 (2.5–5.0) ^§^	NR	47.8% (11/23) 73.9% (17/23)	NR

RCT, randomized controlled trial; WON, walled-off necrosis; LAMS, lumen-apposing metal stent; DPS, double pigtail stent; ^§^ median (range); ^+^ mean (standard deviation); NR, not reported.

### 3.3. Comparison with Other Approaches

However, despite promising evidence from observational studies, RCTs comparing LAMSs and DPSs have failed to demonstrate clear superiority of one technique over the other. The first RCT on this topic, by Bang et al., randomized 61 patients with symptomatic WON to drainage with either LAMSs (*n* = 31) or DPSs (*n* = 29), reporting no significant differences in clinical success rates or overall AE [52]. Similarly, subsequent RCTs and comparative studies found no significant differences in key clinical outcomes, including clinical success, number of necrosectomy sessions, and AE, with only one study reporting higher clinical success at 8 weeks (but not at 4 weeks) in the LAMSs group [53,54,55,56]. Notably, Bang’s study reported an increased risk of stent-related AE, especially clinically significant bleeding, in the LAMSs group during the initial phase of the study [52]. This led to a protocol change recommending earlier LAMS removal (after 4 weeks). The proposed mechanism of bleeding involved rapid collection collapse and subsequent trauma from the LAMS internal flange to retroperitoneal vessels [57]. However, subsequent studies have questioned the true impact of removal timing on AE, suggesting that LAMSs can be safely left in place beyond 4 weeks if clinically necessary [42,58,59].

In conclusion, current evidence suggests that EUS-guided PC drainage with LAMSs is highly effective and generally safe. Optimal outcomes likely depend on accurate patient selection and individualized treatment planning. Given the advantages of simplified stent deployment and stable access for repeated necrosectomy sessions, patients with a high necrotic burden and an anticipated need for EN may benefit the most from this approach [60,61,62,63].

## 4. EUS-Guided Gallbladder Drainage (EUS-GBD)

EUS-guided gallbladder drainage (GBD) with LAMSs has emerged as a minimally invasive and effective alternative for acute cholecystitis (AC) in patients unfit for surgery or at high operative risk [64,65]. Candidates typically include elderly patients (>80 y/o) or those with comorbidities (e.g., cardiopulmonary disease, cirrhosis, or coagulopathy) with ASA ≥ 3 or CCI ≥ 4. While cholecystectomy remains the gold standard, in this population, EUS-GBD can serve as both a definitive therapy or as a temporary bridge to surgery. Importantly, the LAMS access site also permits intraluminal lithotripsy for gallstone removal, using laser or mechanical devices. Moreover, conversion of percutaneous drainage (PT-GBD) to EUS-GBD is another potential application, as well as its use as salvage after failed percutaneous or endoscopic drainage [66].

### 4.1. Procedural Considerations and Stent Placement

**Figure 3:**
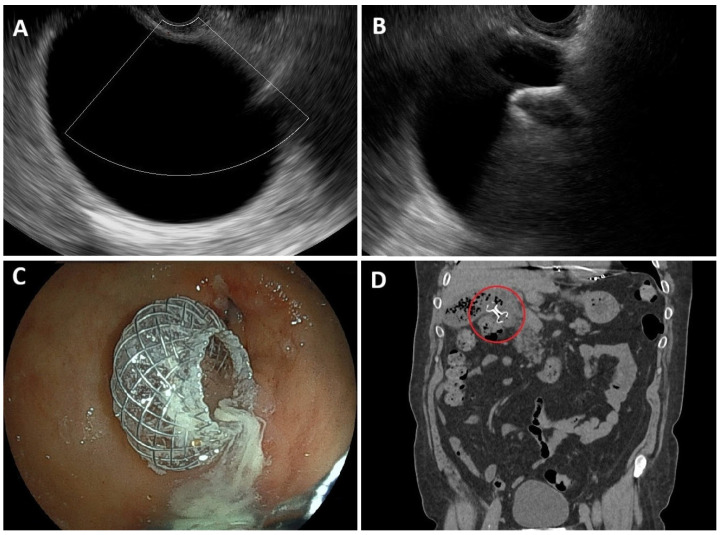
Procedural steps of EUS-guided gallbladder drainage: (**A**): EUS view showing a hydropic gallbladder. (**B**): Deployment of a lumen-apposing metal stent (LAMS) into the gallbladder. (**C**): Endoscopic view demonstrating the transduodenal LAMS in position. (**D**): Abdominal CT scan of a transduodenal LAMS in place (red circle) for gallbladder drainage.

Under EUS guidance, the distended gallbladder is identified while avoiding interposing vessels or large obstructing stones; wall integrity and thickening must be evaluated to rule out gangrenous cholecystitis [67]. The procedure is ideally performed with an anesthesiologist’s support and fluoroscopy [68]. Transmural puncture (usually via gastric antrum or duodenal bulb), guidewire insertion, tract dilation, and stent release are steps traditionally performed (Figure 3). Modern cautery-enhanced LAMSs allow a single-step, freehand deployment, reducing time (2.5 vs. 9 min) and wire manipulation, minimizing AE rates [69,70,71,72]. In fact, the dual-flanged saddle design provides stable anchorage and prevents leakage or dislodgement. A 10 × 10 mm LAMS is most commonly used. International consensus now recommends insertion of a double-pigtail stent within the LAMS in transgastric placement or when stones are present [25].

### 4.2. Outcomes and Safety

EUS-GBD with LAMSs has shown consistently high efficacy. Even if several studies, mostly retrospective, have been conducted (Table 2), a recent systematic review and meta-analysis including 18 studies and 701 high-risk patients with ≥1 year follow-up, reported technical and clinical success rates of 95.8% and 94.3%, respectively. The overall AE rate was ~10%, recurrence of cholecystitis 4.2%, need for repeat endoscopy for stent obstruction/occlusion 2.9%, and mean stent patency 418.8 days, supporting durable LAMS patency, a long-term option with a favorable safety profile. Notably, most complications were manageable endoscopically [73]. Despite the clear advantages of the procedure, many questions remain, among which one of the most relevant is to establish a risk stratification and a decision-making algorithm for EUS-GBD in high-risk surgical patients with AC [66].

### 4.3. Comparison with Other Approaches

PT-GBD, although effective, requires prolonged external catheterization, carries high reintervention rates, and significantly impacts quality of life. Moreover, PT-GBD is linked to multiple catheter-related complications, including stoma site pain, increased infection risk, and catheter [74]. As a bridge to surgery, EUS-GBD was linked to shorter operative times, hospital stay, and earlier cholecystectomy relative to PT-GBD [75]. Although initial costs are higher compared to PT-GBD, reduced readmissions and reinterventions may yield long-term economic advantages for EUS-GBD [76].

Compared with laparoscopic cholecystectomy, a propensity score analysis found equal technical success (100%), clinical success of 93.3% vs. 100%, and similar 30-day AE and reintervention rates [77]. Interval cholecystectomy after EUS-GBD remains substantially feasible, with a pooled success 32.9% and AE rate of 13.2%, mainly using laparoscopic approaches and without procedure-related mortality [78].

In an international RCT, Teoh et al. reported comparable success rates between EUS-GBD and PT-GBD (97.4% vs. 100% and 92.3% vs. 92.5%), but EUS-GBD significantly reduced 1-year and 30-day AE rates, reinterventions and readmissions, recurrent cholecystitis (2.6% vs. 20%, *p* = 0.029). Post-procedural pain scores and analgesic requirements were notably lower in the EUS-GBD group [79].

## 5. EUS-Directed Transgastric ERCP (EDGE) and EUS-Directed Trans-Enteric Endoscopic Retrograde Cholangiopancreatography (EDEE)

EUS-directed trans-gastric endoscopic retrograde cholangiopancreatography (EDGE) and EUS-directed trans-enteric endoscopic retrograde cholangiopancreatography (EDEE) are recently developed procedures that allow the performance of an ERCP in certain surgically altered anatomy (SAA), creating a «shortcut» for easier access to the papilla or to biliary and/or pancreatic anastomosis [23].

EDGE was initially the acronym of a different procedure, performed in 6 patients, were the EUS-guidance was used only for injecting sterile water and air, in order to distend the excluded stomach in patients who had undergone a Roux-en-Y gastric bypass (RYGB) for obesity, and allow a radiological gastrostomy in an easier way, followed by an ERCP through a self-expandable metal stent placed through the gastro-cutaneous fistula. The advent of LAMSs completely changed this procedure and, in 2014 a LAMS was placed directly through the gastric pouch and the excluded stomach allowing an ERCP at the same time [80,81]. The success of this procedure has encouraged the development of this technique for performing an ERCP in others SAA, realizing entero-enteric anastomosis through EUS-guided placement of a LAMS. This technique was initially described in a post-surgical malignant scenario [82] and then in benign conditions [83,84]. The term endoscopic entero-enteral bypass (EEEB), used in the largest published experience, is commonly replaced by the acronym EDEE, first used in 2020 [85], and more similar to the original EDGE acronym, describing the trans-gastric approach.

The advent of electrocautery enhanced delivery systems, represented a breakthrough for the diffusion of gastro-gastric, gastro-enteric and entero-enteric EUS-guided anastomosis through LAMS’s placement, thus allowing any kind of endoscopic interventions (other than an ERCP) using an EDGE/EDEE approach. Those procedures are generally called EUS-directed trans-gastric intervention (EDGI) [86], but the acronym EDEI should be maybe preferred in case of trans-enteric interventions.

All the above-mentioned procedures can be performed in SAA such as RYGB or other complexes bariatric surgery procedures (duodenal switch, bilio-pancreatic diversion etc.), Roux-en-Y gastrectomy or total gastrectomy, pancreatoduodenectomy with or without pylorus preserving, surgical hepatico-jejunostomy and any other SAA not allowing an easy access to the papilla, or to another target region (bilio-digestive or pancreato-digestive anastomosis, or any target for EDGI procedures).

### 5.1. Procedural Considerations and Stent Placement

The European Society of Gastrointestinal Endoscopy (ESGE) has published a recent technical review on interventional EUS [1], where multiple recommendations are given in order to reduce the risk of complication while performing EDGE or EDEE procedures: 250–500 mL of saline, with or without dye/contrast medium, should be instilled under EUS-guidance with a 19 G needle in the excluded stomach/target loop; LAMSs ≥ 15 mm in diameter with an electrocautery-enhanced delivery system (Autocut mode 100–150 W, effect 3–5) should be used for fistula creation, with a preference for 20 mm in diameter LAMSs (as well as a balloon dilation to 15 mm) when considering a same-session ERCP; both gastro-gastric or jejuno-gastric approach could be used for EDGE procedure and it’s important to take care not to deploy the LAMSs too caudally in the antrum or distal gastric body, as this may complicate subsequent insertion of the duodenoscope; a delay of at least 7 days between LAMS placement and ERCP should be observed, whenever possible, in order to allow the LAMSs to fully expand and the fistula to mature; LAMS removal should not be performed within the first 7 days and only when no additional interventions are required; the use of anchoring techniques (to prevent LAMS migration) is controversial and there is no clear indication for persistent fistula closure.

In terms of the deployment of LAMSs in the target lumen over a guidewire (the so-called direct technique over a guidewire, DTOG), it has been found to be associated with an increased risk of adverse events, as compared to the wireless endoscopic simplified technique (WEST) [87], that is a “free hand” placement of the LAMS without guidewire. The ESGE technical review advise a slow withdrawal of the guidewire while performing the DTOG, because the wire can push the target organ further away, possibly leading to stent mal-deployment [1].

Regarding EEEB/EDEE procedures, 4 different techniques were used in a large single center experience [88] for the injection of saline (with contrast medium) in order to identify and distend the target (biliary) loop: (1) percutaneous transhepatic biliary drainage performed before EEEB session and using the percutaneous drain for irrigation; (2) transgastric EUS-guided puncture of the left hepatic duct and injection (in order to opacify and distend the biliary loop); (3) direct EUS-guided puncture of the jejunal loop; (4) placement of a 7 Fr endoscopic tube into the jejunal loop, in a retrograde manner, for irrigation.

### 5.2. Outcomes and Safety

EDGE has been shown to be a cost-effective procedure, as compared to enteroscopy-assisted (EA-ERCP) and laparoscopic-assisted ERCP (LA-ERCP) [89,90].

A major limitation to its routine use in post-RYGB patients is the risk of fistula non-closure. This issue was further evaluated in a multicenter study assessing long-term follow-up and fistula closure outcomes, which included 172 patients [91]. The only variable that predicted fistula persistence was total LAMS indwell time (a mean of 86 days in the persistent group vs. 50 days in the non-persistent group, *p* < 0.004); other variables such as location of LAMS deployment, LAMS diameter, attempted endoscopic fistula closure at LAMS removal, argon plasma utilization (APC) at fistula closure or performance of single-session vs. staged EDGE were not statistically significantly different between the two groups, despite the small sample size; repeat endoscopic procedure to close persistent fistulas was done in 11 of 19 patients, with 100% technical success using a combination of suturing, APC and over-the-scope-clips. In a recent meta-analysis including 16 studies (470 patients), the pooled risk of a persistent fistula was 17% and weight regain was observed in the 4% of patients [92].

Others associated complications are stent migration (7%), bleeding (5%), perforation (4%) and post-ERCP pancreatitis (2%), whereas technical and clinical success are 96% and 91%, respectively, and the adverse event rate is similar to that of EA-ERCP and LA-ERCP, but with shorter procedure time and hospital stay [92].

### 5.3. Comparison with Other Approaches

About EEEB/EDEE results, the larger published study to date is that of Mutignani et al. analyzing 80 patients treated for biliary adverse events (BAE) following surgery with bilio-digestive anastomosis, between 2014 and 2021 in a single center experience: EEEB was technically successful in all but one patient; adverse events (self-limiting bleeding, partial intraprocedural stent displacement, acute respiratory failure, asymptomatic intraprocedural pneumoperitoneum) occurred in 32% of cases; BAE were successfully treated through EEEB in all cases, with a recurrence of 3.8% during the mean follow up of 4 years; recurrences were successfully treated endoscopically, through EEEB [88].

A recent systemic review and meta-analysis of 67 studies (2714 patients) [93] showed a statistical significant difference (*p* < 0.01) of EDGE and LA-ERCP compared to EA-ERCP for accessing the papilla (96% and 93% for EDGE and LA-ERCP, respectively, vs. 77% for EA-ERCP) and successful ERCP (93% and 92% for EDGE and LA-ERCP, respectively, vs. 64% for EA-ERCP). No differences (*p* = 0.49) in complication rates were observed (20% for EDGE, 19% for LA-ERCP and 13% for EA-ERCP).

## 6. EUS-Guided Gastroenterostomy (EUS-GE)

Gastric outlet obstruction (GOO) is a serious complication of pancreatobiliary diseases, typically resulting from mechanical obstruction of the distal stomach (antral and pyloric regions) or the proximal duodenum [94]. The clinical presentation of GOO commonly includes nausea, vomiting, abdominal pain, weight loss, and progressive nutritional deterioration [95]. While GOO is most often caused by malignancies—originating from the stomach, pancreas, ampulla, biliary tract, or duodenum—it may also occur secondary to benign conditions such as acute or chronic pancreatitis, peptic ulcer disease, Crohn’s disease, or strictures from medications or caustic ingestion [96].

EUS-GE using LAMSs has emerged as a cornerstone intervention for malignant GOO in centers with advanced endoscopic expertise [97]. The primary objective of endoscopic management is to bypass the obstruction and restore the ability to tolerate oral intake In this context, robust evidence supports the high technical and clinical success rates of EUS-GE, with a safety profile that is favorable compared to both enteral stenting and surgical gastroenterostomy [97,98,99,100].

To date, only a limited number of studies have investigated the role of EUS-guided gastroenterostomy in the treatment of benign GOO [101]. Traditional endoscopic approaches—such as balloon dilatation or enteral stent placement—have shown inconsistent and often unsatisfactory outcomes in benign settings, with high recurrence rates and limited long-term efficacy. Consequently, surgical intervention remains the standard of care in many cases [94].

More than a decade ago, a case report documented the successful use of EUS-GE for GOO secondary to chronic pancreatitis, marking one of the earliest descriptions of this approach in a benign disease context [102]. Since then, interest has grown in exploring EUS-GE as a minimally invasive alternative to surgery for selected patients with benign GOO.

### 6.1. Technical Steps and Stent Placement

EUS-guided entero-anastomosis is a minimally invasive endoscopic procedure that establishes a bypass between two intestinal loops under EUS guidance. The technique employs a linear echoendoscope to visualize and puncture the target bowel loop—typically the jejunum—from the gastric or proximal intestinal lumen.

Several technical approaches for performing EUS-GE have been described, differing primarily in the methods used to identify and stabilize the target jejunal loop. These can be broadly categorized into device-assisted techniques, such as the EUS-guided double-balloon-occluded gastrojejunostomy bypass (EPASS); direct techniques, which utilize a 19-gauge FNA needle to instill saline mixed with contrast prior to deployment of the electrocautery-enhanced lumen-apposing metal stent (EC-LAMS); and the wireless EUS-GE simplified technique (WEST). In the latter approach, a nasoenteric tube is advanced over a guidewire through the stricture into the small bowel distal to the gastric outlet obstruction. The loop is then distended with saline, and under EUS guidance, the LAMS is deployed using a freehand technique, with the nasobiliary catheter serving as a visual and positional reference.

The instilled saline may be mixed with a contrast medium and/or methylene blue dye to confirm luminal access and enhance fluoroscopic or endoscopic visualization. Adequate distension of the target loop is critical to ensure a stable window for safe puncture and precise anastomosis creation [103].

### 6.2. Outcomes and Safety

Chen Yen-I et al. first published a retrospective multicenter study in 2018, reporting on 26 patients who underwent EUS-GE using LAMSs for the management of benign GOO. Most cases were related to strictures secondary to chronic pancreatitis. The authors reported promising outcomes, with high rates of both technical success (96%) and clinical success (92%), highlighting EUS-GE as a potentially effective and minimally invasive alternative to surgery in selected cases of benign GOO [104].

A recent systematic review and meta-analysis conducted by an American group included ten studies encompassing a total of 181 patients who underwent EUS-GE for benign GOO. The authors reported a pooled technical success rate of 95% and a clinical success rate of 90.6%. Regarding secondary outcomes, the adverse event rate was 11%, and the reintervention rate was 7%, confirming the procedure as a safe and effective alternative to surgery in selected cases of benign GOO [105].

A subsequent and more robust systematic review and meta-analysis conducted by an Italian group included fifteen studies encompassing a total of 376 patients who underwent EUS-GE for benign GOO. The pooled technical success rate was 95.8%, and the clinical success rate was 93.4%. The overall adverse event (AE) rate was 11.6% (95% CI: 6.8–16.5%, I^2^ = 57.18%), confirming the procedure’s high efficacy and acceptable safety profile in benign settings. However, the meta-analysis reported substantial heterogeneity in the AE rate, which was significantly influenced by the methodological quality of the included studies and by variability in AE definitions and classifications, with reported rates ranging from 3% to 17% [101].

### 6.3. Comparison with Other Approaches

Despite the currently limited and heterogeneous evidence in the medical literature regarding the management of GOO secondary to various benign conditions, EUS-GE with LAMS placement has emerged as a promising endoscopic alternative in centers with appropriate expertise. Clinical application of EUS-GE in this context shows technical and clinical success rates comparable to those observed in malignant GOO, with an acceptable adverse event profile when compared to surgical gastroenterostomy.

## 7. Other Emerging EUS-Guided Interventions in Benign Disease

### 7.1. EUS-Guided Entero-Colonostomy and Colo-Colonostomy in Benign Disease

EUS-guided entero-colonostomy and colo-colonostomy are innovative and minimally invasive techniques with a growing field of application in the palliation of malignant disease, demonstrating a high technical (100%) and clinical (83%) success rate [106,107,108]. However, some data are also emerging regarding their use in the management of benign lower gastrointestinal obstruction as a second-line treatment (when dilation, stenting, or surgery are not feasible or have failed). First, in 2018, Mai, H.D. et al. described EUS-guided treatment of small bowel obstruction, which enabled successful management in a high-risk surgical patient, with a short-time discharge achievement [106].

Pang, P.B.C. et al. described a short case series of postsurgical benign complete colorectal anastomosis occlusion, successfully treated with LAMS placement, achieving acceptable anastomotic dilation after stent removal [107]. Although the technique is not well standardized and could require ancillary procedures such as endoscopic dilation, it can offer effective symptom resolution in patients with an extensive surgical history and multiple iatrogenic perforations, which could otherwise exclude them from re-surgical intervention [109].

Although based on small case series, these findings are encouraging, as they demonstrate the feasibility of the procedure with good technical and clinical success and a low rate of periprocedural complications. However, the current evidence is limited by the small number of studies, short follow-up periods, and the off-label use of devices. Consequently, long-term outcomes remain uncertain. Further prospective studies with larger sample sizes and longer follow-up are needed to better define the safety, efficacy, and durability of this technique.

### 7.2. EUS-Guided Biliary Drainage (EUS-BD) for Benign Strictures

ERCP remains the first-line treatment for benign biliary strictures. However, in selected cases, endoscopic ultrasound-guided biliary drainage (EUS-BD) represents a valuable alternative, particularly after failed ERCP attempts. This includes situations where the papilla is inaccessible or in patients with SAA [110,111]. In this context, EUS-guided biliary procedures are primarily performed using an antegrade approach, with the rendezvous technique being the most commonly adopted. These approaches have demonstrated promising technical and clinical outcomes in appropriately selected patients [112,113]. In patients with SAA, EUS-BD is typically performed using hepaticogastrostomy (EUS-HGS) or hepaticojejunostomy (EUS-HJ) approaches. Several small case series have reported promising results, with technical and clinical success rates exceeding 90%, and a low rate of AEs, supporting the feasibility and efficacy of these techniques in this challenging clinical setting [114,115,116,117,118], even considering long-term follow-up, with a recurrence rate of 33% [119].

Regarding the use of LAMSs for benign biliary strictures drainage, literature lacks data and is limited to small case series. Indeed, EUS-guided bilioenteric drainage can be considered in exceptional cases. In those situations, LAMS placement offers a minimally invasive rescue strategy, effectively establishing biliary drainage, maintaining long-term fistula patency, and significantly reducing the need for multiple interventions. Ishikawa et al. described two cases of EUS-guided fully covered self-expandable metal stent (FCSEMS) placement to address recurrent cholangitis after retrograde biliary drainage, by creating a permanent fistula following stent removal [119]. In another report, Iwai et al. described the successful therapeutic use of transluminal EUS-guided choledochoduodenostomy (EUS-CDS) employing a modified FCSEMS to overcome treatment failure after multiple plastic and metal stent placements in patients with chronic pancreatitis-associated biliary strictures [120]. Lastly, this technique was also reported as a rescue treatment after postsurgical choledochoduodenostomy stricture, aimed to calibration stricture [121].

EUS-guided LAMS placement finds its greatest application in SAA, allowing for the creation of an entero-enteral bypass and thereby increasing both technical and clinical success rates when deep cannulation cannot be achieved through retrograde approach [122]. According to the experience of Mutignani M. et al., in a tertiary referral center, the entero-enteral endoscopic by-pass creation lead to highly successful treatment of benign biliodigestive anastomotic strictures, with a 32% rate of AEs and low recurrence rate over 7 years [83].

However, in this setting, the use of LAMSs is primarily intended to facilitate access to the biliary limb and is therefore only indirectly associated with biliary drainage. Moreover, as previously mentioned, data on the use of LAMSs for direct biliary drainage remain inconsistent, particularly regarding long-term clinical outcomes.

## 8. Conclusions

EUS-guided drainage has become an indispensable tool in managing benign pancreatobiliary diseases. With advancements in stent technology, particularly LAMSs and EC-LAMSs, and a better understanding of patient selection and procedural planning, these minimally invasive procedures offer a promising alternative to traditional surgical interventions, leading to improved patient outcomes and fewer complications.

However, current evidence largely derives from small case series conducted at high-volume centers, an inherently biased study design that limits the generalizability of some reported outcomes. Moreover, substantial heterogeneity in institutional expertise persists and may significantly influence the selection of the most appropriate therapeutic approach. Importantly, the paucity of long-term follow-up data underscores the need for well-designed, high-quality studies to establish the safety, efficacy, and durability of LAMSs across the spectrum of benign pancreatobiliary and gastrointestinal diseases discussed in this review.

As the field continues to evolve, ongoing innovations will further refine these techniques and broaden their indications.

## Figures and Tables

**Table 2:** Main studies about EUS-GBD outcomes in high-risk surgical patients with acute cholecystitis.

First Author Year	Study Design	Patients Number	Technical Success	Clinical Success	Adverse Events
Walter, D. et al. (2016) [4]	Multicenter, prospective	30	90%	96%	AC recurrence 7% AEs 50% (stent-related or procedure-related—13%) Overall mortality 23% (30-day mortality—17%)
Teoh, A.Y.B. et al. (2020) [74]	Prospective, multicenter open labelled RCT	39	97.4%	92.3%	AC recurrence 2.6% AEs 25.6% (at 1 year) AEs 12.8% (at 30 days) Re-interventions 2.6% (at 30 days) Unplanned readmissions 15.4%
Martinez-Moreno et al. (2023) [18]	Single center, retrospective	50	100%	98%	AC recurrence 4%. AEs 18%, 20%, and 26% at first, second, and third years of follow-up. Stent migrations 14% Symptomatic LAMS-related AEs 37.5% No stent-related bleeding or stent-related mortality
Inoue et al. (2023) [72]	Single center, retrospective	90	96.7%	91.9%	AC recurrence 3.8% AEs 5.0% Longer time to late AEs
Binda et al. (2024) [26]	Multicenter, retrospective	40	95%	92.5%	No AC recurrence (follow-up > 1 year) AEs 10.3% (no fatal AEs).
Chon, H.K. et al. (2024) [71]	Single center, retrospective	58	94.8%	100%	AC recurrence 3.6% Early AEs 1.8% (stent obstruction) Late AEs 5.4% (cholangitis + stent obstruction)

AC, acute cholecystitis, AE, adverse event; RCT, randomized controlled trial; LAMS, lumen-apposing metal stent.

## Data Availability

The original contributions presented in this study are included in the article. Further inquiries can be directed to the corresponding author.

## Abbreviations

The following abbreviations are used in this manuscript:

EUS	endoscopic ultrasound
LAMS	lumen-apposing metal stents
ERCP	endoscopic retrograde cholangiopancreatography
EC	electrocautery
PC	pancreatic collections
PP	pancreatic pseudocysts
WON	walled-off necrosis
DPS	double-pigtail stents
EN	endoscopic necrosectomy
AE	adverse events
RCT	randomized clinical trials
EUS-GBD	EUS-guided gallbladder drainage
AC	acute cholecystitis
PT	Percutaneous drainage
EDGE	EUS-directed trans-gastric endoscopic retrograde cholangiopancreatography
EDEE	EUS-directed trans-enteric endoscopic retrograde cholangiopancreatography
SAA	surgically altered anatomy
RYGB	Roux-en-Y gastric bypass
EEEB	endoscopic entero-enteral bypass
EDGI	EUS-directed trans-gastric intervention
ESGE	European Society of Gastrointestinal Endoscopy
DTOG	direct technique over a guidewire
WEST	wireless endoscopic simplified technique
EA-ERCP	enteroscopy-assisted
LA-ERCP	laparoscopic-assisted ERCP
APC	argon plasma coagulation
BAE	biliary adverse events
GOO	gastric outlet obstruction
EUS-GE	EUS-Guided Gastroenterostomy
EPASS	EUS-guided double-balloon-occluded gastrojejunostomy bypass
EUS-BD	endoscopic ultrasound-guided biliary drainage
EUS-HGS	endoscopic ultrasound-guided hepaticogastrostomy
EUS-HJ	endoscopic ultrasound-guided hepaticojejunostomy
FCSEMS	fully covered self-expandable metal stent
